# Single ventricle with persistent truncus arteriosus as two rare entities in an adult patient: a case report

**DOI:** 10.1186/1752-1947-2-184

**Published:** 2008-05-30

**Authors:** Inna Porter, James Vacek

**Affiliations:** 1 University of Kansas Hospital, Rainbow Boulevard, Kansas City, KS 66160, USA

## Abstract

**Introduction:**

Single ventricle and truncus arteriosus are both rare congenital cardiac syndromes with limited survival. Their occurrence together is extremely uncommon and prolonged survival is exceptionally rare. We present the case of a patient who had both of these defects with survival to age 45.

**Case presentation:**

We describe the vase of a 45-year-old man with the unusual occurrence of two very rare congenital cardiac defects. He was found to have both truncus arteriosus and single ventricle with long survival. His history, clinical course, and anatomic findings are discussed along with the factors which may have contributed to his longevity, which is unique in the medical literature. His management reflected the state of medical knowledge at the time when he presented, and although alternate approaches may have been utilized if the patient presented today, this case does indicate the efficacy of the management options available at the time and place of the patient's contacts with the medical care system in Belarus. We discuss the findings, frequency, classification, and management of both of these congenital defects.

**Conclusion:**

This case demonstrates that patients with very complex congenital cardiac disease may survive to adulthood, presenting challenges in both medical and surgical treatment. As the management of these patients is constantly evolving, and interventional techniques are improving, patients such as this with prolonged survival will be more common, with each case providing insights to future treatment. Challenges in management may include prior care provided in health care systems with limited resources.

## Introduction

We present the case of a patient who was born with the simultaneous occurrence of two congenital cardiac defects, truncus arteriosus and single ventricle, which are individually uncommon. The patient survived to age 45, which has not been reported previously for a patient with these types of defects. We discuss this patient's medical history, physical, laboratory, and autopsy findings, and provide a review of the individual congenital cardiac lesions. We include a literature review for the congenital cardiac defects as well as the results of investigation for similar prior cases [1-19] which discusses anatomic findings, occurrence, management, and outcomes.

## Case presentation

A 45-year-old man presented to our hospital in Belarus with the following history. He had been born in Belarus after an uncomplicated pregnancy. His family history was negative for congenital defects and the patient had no siblings. After birth, a systolic murmur was heard along the left sternal border leading to a referral to a pediatric cardiology centre in Ukraine where the diagnosis of tetralogy of Fallot was given based on physical examination, chest radiographs, and an electrocardiogram. Surgery was declined at that time. At no time in the patient's life were chromosomal studies undertaken. The patient had no children.

As a child, the patient had normal growth and mental development, but marked cyanosis, weakness, clubbing, and intolerance of moderate physical activity. The patient was referred to a medical institute in Moscow at age 18, after several episodes of syncope. A diagnosis of severe pulmonary stenosis with ventricular septal defect was considered at that time. A right Blalock-Taussig shunt was performed. The postoperative diagnosis was single ventricle type BIII with severe pulmonary artery stenosis and hypoplasia. After surgery, the patient was treated with digoxin, pentoxifylline, and spironolactone. The patient's condition improved significantly and he was able to walk several blocks without significant dyspnea.

The patient's condition remained stable for the next five years. He had shortness of breath with moderate exertion, but he was asymptomatic at rest. At 27 years of age, the patient reported an increase in dyspnea with minimal exertion. He was diagnosed with thrombosis of the Blalock-Taussig anastomosis and was treated with heparin for 4 weeks. He never returned to his improved, postoperative condition. He complained of palpitations, dull chest pain at rest, episodes of shortness of breath at rest, and abdominal pain. The patient had several documented episodes of ventricular tachycardia at age 35 years and was successfully treated with propafenone.

At 39 years of age, the patient presented to our hospital in Belarus. At the time of presentation, he complained of severe cyanosis, shortness of breath with minimal exertion, and chest pain. An electrocardiogram indicated sinus tachycardia of 120 beats per minute. The QRS axis was to the right (mean axis +130°). High QRS voltage suggestive of ventricular hypertrophy was noted. There were also ventricular extrasystoles in a trigeminal pattern and horizontal ST-segment depression in the inferior leads. A 24-hour cardiac monitor showed multiple episodes of non-sustained ventricular tachycardia with subjective feelings of palpitation and lightheadedness.

The patient was switched empirically from propafenone to mexiletine with better control of his ventricular tachycardia. His hematocrit was 58% to 64%. Transcutaneous oxygen saturation was 75% to 85% on room air. The patient could tolerate well most of his daily activities such as walking for two blocks, grocery shopping, and performing minor work at home. Over the next 6 years he had repeated hospitalizations for dyspnea, chest pain, and near syncope. He was treated with phlebotomies, saline and/or dextran infusions to improve viscosity, and medications including spironolactone, pentoxifylline, and aspirin.

**Figure 1:**
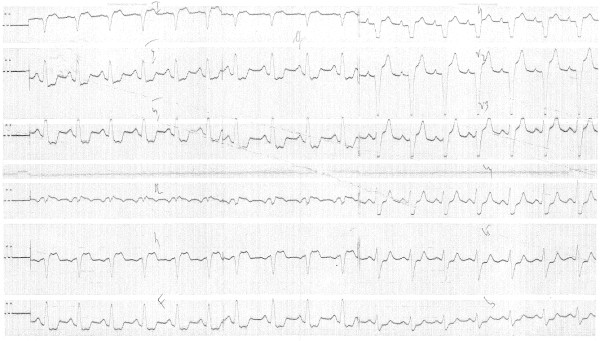
**Electrocardiogram from the terminal hospitalization**. Leads are as follows: top to bottom on the left, I, II, III, aVR, aVL, aVF; and top to bottom on the right, V1 through V6.

**Figure 2:**
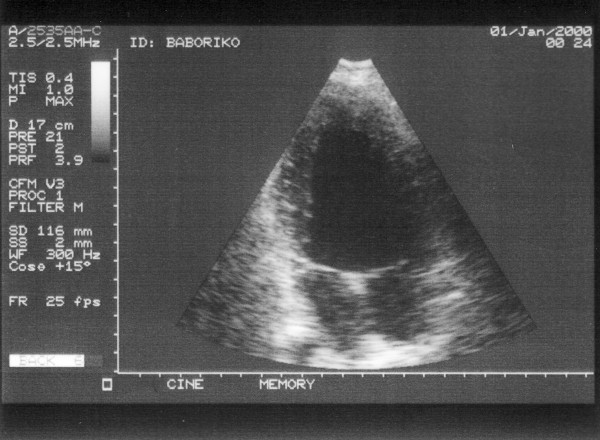
**Echocardiogram from the terminal hospitalization**. Apical view showing single ventricle with two atrioventricular valves.

At the time of the terminal hospitalization, physical examination was remarkable for a 3/6 systolic murmur heard at the upper left sternal border, edema of the right ankle and foot, and tachycardia of 140 beats per minute. The patient was noted to experience severe shortness of breath with even minimal exertion and was deeply cyanotic. The electrocardiogram is shown in Figure 1 and demonstrated sinus rhythm with a mild tachycardia at a rate of 110 beats per minute, right axis deviation, and a non-specific intraventricular conduction delay with diffuse ST-T changes. Chest radiograph indicated a small pleural effusion on the right and increased pulmonary vascularity. The heart was moderately enlarged with prominence of the aorta. An echocardiogram was performed (Figure 2). A single ventricle with unremarkable atrioventricular valves was seen. The end diastolic diameter of the ventricle was measured as 69 mm, the posterior wall thickness was 17 to 19 mm, and the left atrium measured 29 mm. The estimated ejection fraction was 45% to 50%. A vessel with a semilunar valve (the truncal valve) arising from the ventricle was seen. The cusps of the valve were hyperechogenic. Moderate regurgitation was noted. No pulmonary artery was seen. Doppler study of the lower extremities showed thrombosis in the veins of the right calf. Heparin therapy and intravenous fluids were initiated. The patient's condition deteriorated rapidly and several hours later he became comatose and died.

Autopsy showed an enlarged heart that weighed 750 g, composed of two atria with an intact septum, and a single ventricle. The right atrium was enlarged to a diameter of 10 cm. The diameter of the left atrium was 3 cm. Both caval veins emptied normally into the right atrium. All four pulmonary veins entered the left atrium normally. Both atrioventricular valves had normal anatomy, free of vegetations. The single ventricle with left ventricular characteristics had a diameter of 12 cm with a wall thickness of 2 to 2.2 cm. A single artery, truncus arteriosus, arose from the ventricle and arched to the left. The truncal valve had three cusps that were moderately calcified. The coronary ostia and vessels were normal. The pulmonary artery trunk was located 3.5 cm from the origin of the truncus and divided to form left and right pulmonary arteries. At the hilum of the right lung the right pulmonary artery was surgically connected to the right subclavian artery. Red-gray masses were noted at the Blaylock-Taussig shunt anastomosis. Below the anastomosis the right pulmonary artery was almost completely occluded by a dark red adherent thrombus. The ligamentum arteriosum was a fibrous cord. Microscopically the lungs showed dilatation of the pulmonary arterioles and alveolar capillaries. Many bronchial and pulmonary arterioles contained recanalized thrombi or emboli.

The main anatomic diagnoses were: single ventricle with truncus arteriosus; status post Blalock-Taussig procedure; old thromboses of the established anastomosis; hypoplasia of the right pulmonary artery; congestive heart failure; recent thrombosis of the right pulmonary artery; and deep vein thrombophlebitis of the right calf.

## Discussion

A single ventricle is defined as a heart with one ventricle receiving inflow from two separate atrioventricular valves or a common atrioventricular valve [1]. Single ventricle accounts for about 1% of all cardiac anomalies with an incidence of about 0.05 to 0.1 per 10,000 live births [2]. The cause is unknown, but it is most likely multifactorial with a genetic predisposition.

Single ventricles may be classified based on the location of the great arteries [3]. There may be normally related great arteries (type I), D-transposition of the great arteries (type II), or L-transposition (type III). The existence of pulmonic stenosis or pulmonary atresia further subdivides the types of single ventricle. A single ventricle may be accompanied by pulmonary atresia (type A), presence of pulmonic stenosis (type B), or absence of pulmonic stenosis (type C). Depending on the ventricular morphology, the single ventricle can be subdivided as left ventricular type (65% to 70%), right ventricular type (20%), or indeterminate type (10% to 14%) [3]. All patients with a single ventricle have some degree of hypoxemia caused by intracardiac shunting. Clinical manifestations are usually apparent shortly after birth. The most common findings are dyspnea, tachycardia, cyanosis, and progressive heart failure. Later, secondary erythrocytosis and clubbing are usually present.

The diagnosis may be defined by echocardiography, cardiac catheterization, and cardiac magnetic resonance imaging. Treatment options depend on the presence of associated defects. Infants with increased pulmonary blood flow and pulmonary artery pressure require pulmonary artery banding. This procedure helps to prevent early death from congestive heart failure but carries a significant surgical mortality rate. Patients with severe pulmonary outflow obstruction require creation of an aortopulmonary shunt. The Blalock-Taussig procedure is the most commonly performed shunting operation. A modified Fontan procedure may be performed later to separate the pulmonary and systemic circulations. Operative mortality is about 8% to 25% and 10-year survival is 60% to 81%, depending on the pre- and postoperative risks [4]. The median life expectancy of patients without surgical correction is 4 to 14 years [5], although there are descriptions of very rare cases in the literature when such patients have survived over 40 years [2,5-8]. About 65% to 75% of patients without surgical corrections die during the first year of life [2]. The most common causes of death are arrhythmias, heart failure, and sudden cardiac death.

Truncus arteriosus is another rare anomaly, defined as a single great artery that originates from the base of the heart and gives rise to the pulmonary, systemic, and coronary circulation [9-13]. A single semilunar valve is found in truncus arteriosus. The arterial trunk can be connected with the right ventricle, left ventricle, or override and be symmetrically distributed over both ventricles. It has an incidence of about 0.5 to 0.9 per 10,000 live births [9]. A ventricular septal defect is almost always present. Several classifications are used for this anomaly. Van Praagh [10] classified the disorder as types A and B. In type B, there is no association with ventricular septal defect. Type A is subdivided as follows:

1. Type A1: partially separated pulmonary trunk.

2. Type A2: two pulmonary arteries arising directly from the truncus arteriosus.

3. Type A3: a single pulmonary artery originating from the arterial trunk, along with collaterals originating from the descending aorta.

4. Type A4: significant abnormalities of the aortic arch in association with anomalies of the ductus arteriosus.

Patients with truncus arteriosus have some degree of cyanosis during the first week of life. Congestive heart failure usually occurs by a few weeks of age. Excessive pulmonary blood flow at high pressure results in pulmonary vascular obstructive disease by 3 months. The diagnosis of truncus arteriosus is suspected in newborns with mild cyanosis, a cardiac murmur, and pulmonary overcirculation. Factors that limit pulmonary blood flow, such as pulmonary artery stenosis or persistently elevated pulmonary vascular resistance, may delay the appearance of symptoms. The diagnosis is established by echocardiography and cardiac catheterization. The only definitive treatment for this anomaly is surgical correction. Complete repair is preferred and involves three major components: separating the pulmonary arteries from the main truncus, closure of the ventricular septal defect using a patch, and creating a connection between the right ventricle and the pulmonary arteries using a valve conduit, usually a homograft pulmonary artery. Currently over 90% of children survive repair of truncus arteriosus. Long-term survival after surgical correction is about 83% at 15 years after surgery [10]. The prognosis for patients without surgical correction is dismal. The mortality rates are about 20% at 1 week of age and more than 90% at 1 year [11].

Our presented case is a unique case of a single ventricle with truncus arteriosus type A1 (Van Praagh classification) in a patient who lived for 45 years. The reported association of these two defects is extremely rare [6,14-19], and most of these patients die within a few weeks of birth. As described above, the embryology of a single ventricle and truncus arteriosus is different. It is thought that the unlikely concurrence of the two unusual developmental defects within the same patient may explain the extreme rarity of this condition.

The prolonged survival of the presented patient is also exceptional. Only one case of long-term survival of a patient with a single ventricle defect and truncus arteriosus has been reported previously [6] which was a woman with a single ventricle and truncus arteriosus (apparently type 4) who lived for 56 years. To the best of the authors' knowledge there are no cases of prolonged survival reported for a patient with a single ventricle and type 1A truncus arteriosus. The patient in our report had a history of pulmonary stenosis and pulmonary hypoplasia (not typical for truncus type 1) that limited pulmonary flow transmission of systemic pressure to the pulmonary arterial system and likely improved his survival. In patients with single ventricle who do not have significant obstruction to pulmonary arterial flow with protection of the pulmonary vasculature, early occurrence of congestive heart failure or pulmonary vascular occlusive disease is likely with very limited survival unless a procedure is undertaken to limit pulmonary flow (such as pulmonary banding). If, however, severe pulmonary stenosis or atresia is present without provision of adequate pulmonary flow by surgical intervention with a shunt procedure, outcomes are also very poor due to severe cyanosis. Survival is optimized when an appropriate balance of systemic to pulmonary flow is present either spontaneously or by surgical intervention. In this patient, his initial degree of pulmonary outflow obstruction and subsequent Blalock-Taussig shunt fortunately provided the necessary achievement of a balance of systemic versus pulmonary circulatory flow that allowed prolonged survival.

With the passage of time and advances in medical knowledge and experience, future cases such as this may benefit from other types of surgical interventions, long-term full intensity oral anticoagulation, or different approaches to anti-arrhythmic management. For the patient in this report, placing him on therapeutic anticoagulation after his thrombotic event at age 27 would have been a strong consideration. Anticoagulation in the setting of prior thromboembolic events or significant polycythemia for a patient such as this is very reasonable. Utilization of thrombolytic therapy in the setting of a suspected life-threatening thromboembolic event should also be considered, although diagnosis may be difficult.

Management of arrhythmias in patients such as this, as for many congenital heart disease patients, remains challenging with limited data in subsets of unusual entities such as those expressed by our patient [20]. Options for management include typical anti-arrhythmic agents (amiodarone, mexilitine, beta blockers), investigational agents, radiofrequency ablation, or implantation of cardioverter-defibrillators. Much of the available literature on radiofrequency ablation in patients with congenital heart disease relates to supraventricular arrhythmias, without clear documentation of mortality benefit, but symptomatic improvement in some patients [21]. Due to the complexity of anatomy, both intrinsic and corrected, identification and radiofrequency ablation of arrhythmic foci may be difficult. Utilization of an implanted device may be best reserved for patients with prior episodes of sudden cardiac death, documented sustained ventricular tachycardia, unexplained syncope, and/or significantly reduced ventricular function, although definitive documentation of benefit is lacking at this point in time [22].

## Conclusion

In summary, we have presented a unique case of a man who lived for 45 years with a single ventricle with truncus arteriosus type I. The described association of these two defects is extremely rare and the prolonged survival of this man is also exceptional. To the best of the authors' knowledge there are no cases of prolonged survival reported in a person with a single ventricle and type 1 truncus arteriosus.

## Competing interests

The authors declare that they have no competing interests.

## Consent

Written informed consent could not be obtained in this case since the patient's next-of-kin were untraceable. We believe this case report contains a worthwhile clinical lesson which could not be as effectively made in any other way. We expect the patient's next-of-kin not to object to the publication since every effort has been made so that the patient remains anonymous.

## Authors' contributions

IP was involved with the patient's care at the end of his life as well as gathering the case history, doing the majority of the literature review, and writing the original manuscript.

JV has reviewed and revised the manuscript through several drafts, extensively modified substantial portions of the narrative, and performed some of the literature review.
